# Effects of the Racket Polar Moment of Inertia on Dominant Upper Limb Joint Moments during Tennis Serve

**DOI:** 10.1371/journal.pone.0104785

**Published:** 2014-08-12

**Authors:** Isabelle Rogowski, Thomas Creveaux, Laurence Chèze, Pierre Macé, Raphaël Dumas

**Affiliations:** 1 Université de Lyon, Université Claude Bernard Lyon 1, Centre de Recherche et d'Innovation sur le Sport - EA 647, UFRSTAPS, Villeurbanne, France; 2 Université de Lyon, Université Claude Bernard Lyon 1, IFSTTAR, UMR_T9406, LBMC Laboratoire de Biomécanique et Mécanique des Chocs, Bron, France; 3 Babolat VS, Lyon, France; VU University Amsterdam, Netherlands

## Abstract

This study examined the effect of the polar moment of inertia of a tennis racket on upper limb loading in the serve. Eight amateur competition tennis players performed two sets of 10 serves using two rackets identical in mass, position of center of mass and moments of inertia other than the polar moment of inertia (0.00152 vs 0.00197 kg.m^2^). An eight-camera motion analysis system collected the 3D trajectories of 16 markers, located on the thorax, upper limbs and racket, from which shoulder, elbow and wrist net joint moments and powers were computed using inverse dynamics. During the cocking phase, increased racket polar moment of inertia was associated with significant increases in the peak shoulder extension and abduction moments, as well the peak elbow extension, valgus and supination moments. During the forward swing phase, peak wrist extension and radial deviation moments significantly increased with polar moment of inertia. During the follow-through phase, the peak shoulder adduction, elbow pronation and wrist external rotation moments displayed a significant inverse relationship with polar moment of inertia. During the forward swing, the magnitudes of negative joint power at the elbow and wrist were significantly larger when players served using the racket with a higher polar moment of inertia. Although a larger polar of inertia allows players to better tolerate off-center impacts, it also appears to place additional loads on the upper extremity when serving and may therefore increase injury risk in tennis players.

## Introduction

Since the 1980s, the physical characteristics of the tennis racket have been changed drastically by modern designs and materials. The most obvious of the substantial changes in the racket frame is an increase in head size, which accommodates more efficient off-center impacts [1]. To control for advancements in racket design, the international Tennis Federation limited the frame size, i.e. length and racket head area [2]. Nowadays, alterations in the racket's inertial parameters are mainly obtained by manually adding mass to specific locations on the frame [3].

**Figure 1:**
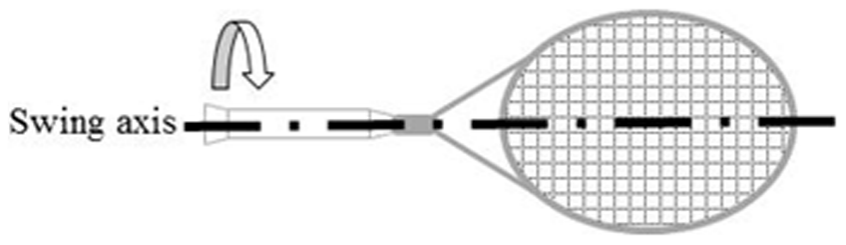
The polar moment of inertia of a tennis racket.

An overly light racket may be counterproductive as the transfer of energy to the ball becomes less effective [2]; but deliberate distribution of mass allows the maneuverability, power and control specifications of the racket to be modified [3]. Indeed, adding mass in the handle results in a racket that is easier to swing rapidly than when mass is added at the tip of the frame [3]. Adding mass symmetrically on both sides at mid-height of the racket head increases the polar moment of inertia (Figure 1) and the racket becomes more resistant to the long-axis twisting motions that occur when the ball is impacted on the lateral portions of the racket face [1]. Therefore, players who find it difficult to consistently impact the ball in the center of the racket face may be able to increase the efficiency of their racket-ball collision by using rackets with larger polar moments of inertia [2]. In addition, the increase in game speed reduces the time available for players to intercept the ball and execute groundstrokes, thus increasing the likelihood of off-center impacts. As a consequence, an increased polar moment of inertia may counteract the reduction in ball control that accompanies suboptimal impact locations. However, there is a paucity of information on the effects of racket specifications, in particular increased racket polar moment of inertia, on upper limb joint loads under playing conditions.

Creveaux at al. [4] found no influence of polar moment of inertia on upper limb loads during the tennis forehand drive. There may be several reasons for this. During ground strokes, the elbow and wrist joints are stabilized by upper arm and forearm muscles, and the upper arm appears as a rigid extension of the racket [5]. Conversely, the tennis serve motion requires extensive shoulder external rotation at the end of the backswing [6], followed by rapid shoulder internal rotation, elbow pronation [7], [8] and wrist flexion during the forward swing, and shoulder horizontal flexion and adduction during the follow through [9]. Such large ranges of motion, combined with the high velocities observed during the tennis serve, increase the stress on the upper limb. Dynamic analysis shows excessive joint dynamics [6], notably, peak moment values in excess of 50 N.m [10], [11]. Such loads applied on the upper limb joints are considered components of injury risk, in particular when the motion is repeated frequently [10]. Tennis players play approximately 60 matches per year and hit between 50–150 serves per match, without counting serves during training sessions [6]. Although an increased polar moment improves racket-head stability during off-center impacts (especially for ground strokes), the added inertia also makes it more difficult to rotate the racket along its long axis during the serve. Given that the tennis serve is a potentially injurious stroke, changes in the racket polar moment of inertia could be a factor in upper limb joint overloads during the tennis serve.

The aim of this study was therefore to compare upper limb joint loads from serves using two different rackets, distinguished by their polar moment of inertia. It was hypothesized that increased polar moment of inertia of the tennis racket would lead to increased upper limb joint loads during the serve motion.

## Methods

Eight male tennis players (mean ± SD: age  = 26.7±4.9 years; height  = 1.79±0.04 m; mass  = 76.5±6.6 kg; skill  =  International Tennis Number 3) participated in this study, which was approved by the ethical committee 'Sud-Est II' (Lyon, France). All participants employed a full backswing as part of their service action and gave their written informed consent to participate in this study. No player reported having experienced an injury in the six-month period prior to testing. According to the International Tennis Federation (2004, http://www.tennisplayandstay.com/media/131802/131802.pdf), ITN 3 describes a player who “has good shot anticipation and frequently has an outstanding shot or attribute around which a game may be structured. He can regularly hit winners and force errors off short balls, and can put away volleys and smashes and has a variety of serves to rely on.”

After warming-up, each player performed two sets of ten serves on an indoor acrylic tennis court, randomly using two rackets (noted I_L_ and I_H_). The polar moment of inertia of both rackets was adjusted by adding mass (Scotch Brand Tape Core Series 591, 3 M Company, St. Paul, USA) to appropriate parts of the racket frame. After the rackets were strung (250 N), grip tape applied and video markers pasted on the racket frame, the polar moment of inertia was 0.00152 kg.m^2^ for I_L_ and 0.00197 kg.m^2^ for I_H_; both rackets were identical in mass (0.327 kg), position of center of mass (0.336 m) and swingweight moment of inertia (0.0339 kg.m^2^). The specifications of the racket were measured using a Racket Diagnostic Center (Babolat VS, Lyon, France). I_L_ was typical of the rackets used by male amateur competition tennis players, corresponding to Babolat Drive Z Lite, while I_H_ raised the polar moment of inertia beyond that of commercially available rackets. Each player was instructed to hit flat serves, i.e. with a minimal amount of spin, from the deuce service court with similar post-impact ball velocity and T-direction for both rackets. A radar gun (Stalker Pro II, Stalker Radar, Plano, TX, USA) was placed two meters behind the player at a height of 1.70 m, to measure the ball velocity after impact and give the player feedback on ball speed.

**Figure 2:**
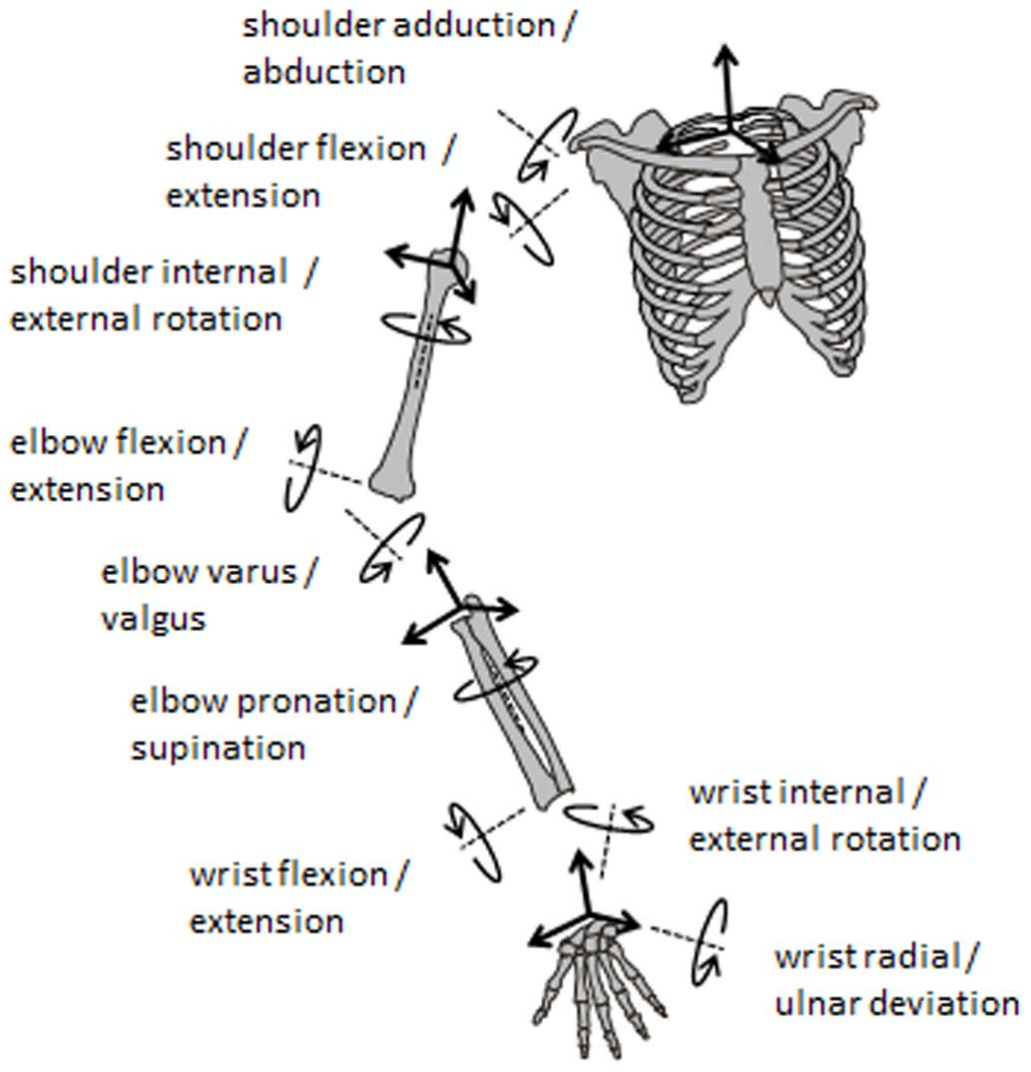
Convention for dynamic measurements.

Fourteen spherical reflective markers (16 mm diameter) were attached to the player and racket to define the segment coordinate systems of the thorax, upper arm, forearm, hand and racket. The markers were affixed to the xiphoid process, incisura jugularis, 7^th^ cervical vertebra, 8^th^ thoracic vertebra, and, on the dominant side, angulus acromialis, medial and lateral humeral epicondyles, radial and ulnar styloid processes, and 2nd and 5th metacarpal heads, as recommended by the International Society of Biomechanics [12]. Two markers were placed symmetrically on both sides at mid-height of the racket head to determine the center of the racket face and one marker was placed on the top end of the handle to define the longitudinal axis of the racket [13]. Retro-reflective tape was placed around the ball to detect the ball–racket impact [13]. Two additional markers were located on the non-dominant shoulder and wrist to detect the beginning of the serve, defined as when the height of the wrist marker was higher than that of the shoulder marker. An eight-camera Eagle motion analysis system (Motion Analysis Corp., Santa Rosa, CA, USA) collected the 3D trajectories of markers during serves at a sampling rate of 500 Hz. Net joint moments and powers were computed as described by Creveaux et al. [13]. Briefly, the net joint moments were computed by a 3D inverse dynamic method [14]. Inputs for the computation were upper limb kinematics, mass, position of center of mass and matrix of inertia of each body segment [15] and the racket. Segment coordinate systems of each upper limb segment were constructed according to International Society of Biomechanics recommendations [12]. Outputs of the computation were the net joint moments acting at the three upper limb joints (wrist, elbow, shoulder) as well as the body segment angular velocities in the global coordinate system. The joint powers were computed as the dot product of net joint moments and the difference between distal and proximal segment angular velocities. The net joint moments were then expressed using the joint coordinate systems [16]. In other words, the net joint moments were projected on the same axes as the axes of the Euler angles computation (Figure 2). The sequences were XZ'Y'' for the shoulder [17], ZX'Y'' for the elbow [12] and ZY'X'' for the wrist joint [18]. To facilitate comparison between the two rackets, the peak of net joint moments and joint powers were normalized. They were divided by the product of player body weight by height, and then multiplied by 100 [10].

For each racket, the three serves with the most similar post-impact ball speed and that landed in the serve box were used for subsequent analysis. For the purpose of this study, the serve was divided into three phases [7], [19]. The cocking phase began at the ball toss and lasted until the maximal shoulder external rotation was reached. The forward swing initiated from maximal shoulder external rotation to the frame prior to impact (−0.002 s); the follow-through was from the frame after impact (+0.002 s) to the racket lowest height after impact. The impact frame was determined according to Creveaux et al. [13]. For the statistical analysis, the normalized moment extrema on each rotation axis of each joint, as well as the mean negative and positive powers at each joint, were extracted during each phase of the tennis serve, yielding 72 parameters characterizing the joint loads. First, to detect potential significant differences related to racket characteristics, multivariate analyses were performed. Twelve datasets composed of 16 lines (8 players * 2 rackets) and six columns (peak normalized moment of both orientations on each rotation axis and mean negative and positive power of one joint for all phases of the serve) were built. Then, Principal Component Analyses (PCA) [20] were performed to determine which variables (dynamic parameters) in each dataset contributed to the main principal components calculation, and how the rackets were located relative to the main principal components. Finally, paired t-tests were performed to test for significant differences between rackets in each parameter selected from the PCAs. Where statistical differences were found, effect sizes for pairwise samples (*d*) were calculated and interpreted according to Cohen's scale [21]. All statistical tests were performed using software SPSS 11.0.1. (Chicago, IL, USA), and level of significance was set at p≤0.05. The values for pre-impact racket resultant velocity, post impact ball velocity, phase duration, and peak normalized moments, as well as mean negative and positive shoulder, elbow, and wrist joint normalized powers during each phase of the tennis serve for both rackets are presented as mean ± standard error (SE).

## Results

The mean pre-impact racket resultant velocities were 22.1±0.4 m.s^−1^ for racket I_L_, and 21.8±0.6 m.s^−1^ for I_H_, and the mean post-impact ball velocities were 37.5±1.4 m.s^−1^ for both rackets. No significant differences between rackets were observed for either velocity. The duration of the three phases did not significantly differ between rackets. On average, the duration was 0.97±0.04 s for the cocking, 0.09±0.01 s for the forward swing, and 0.23±0.01 s for the follow-through of the serve.

**Figure 3:**
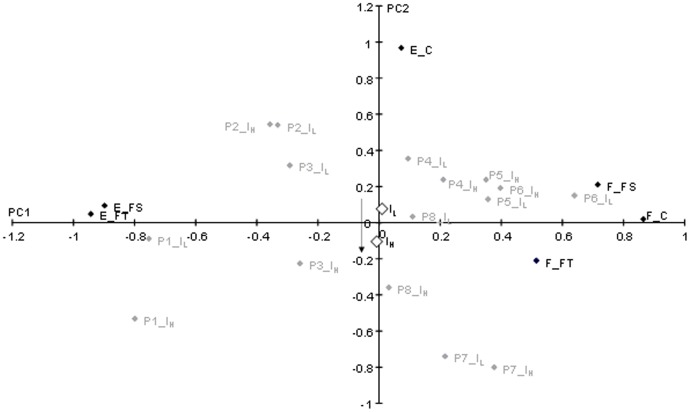
Principal Component 1 (PC1)/Principal Component 2 (PC2) axis system diagram of the peak shoulder extension (E_)/flexion (F_) normalized moments (black markers) during the cocking (C), forward swing (FS) and follow-through (FT) phases. PC1 explains 54% of the dataset variance and PC2 17%. The individuals are represented by grey markers with PX_I_L_ and PX_I_H_ (X ranged from 1 to 8 according to each player), and the supplementary individuals, summarizing the behavior of I_L_ and I_H_, are represented by white markers, I_L_ being the racket with a low polar moment of inertia and I_H_ the racket with a high polar moment of inertia. The arrow indicates the main orientation from I_L_ to I_H_.

From the Principal Component Analysis performed on the twelve datasets (shoulder flexion/extension, shoulder adduction/abduction, shoulder internal/external rotation, shoulder positive/negative power, elbow flexion/extension, elbow varus/valgus, elbow pronation/supination, elbow positive/negative power, wrist flexion/extension, wrist radial/ulnar deviation, wrist internal/external rotation, and wrist positive/negative power; see File S1 for the raw data), only the results of the peak shoulder flexion/extension moments for the three phases of the serve (Figure 3) are presented in detail here (see File S2 for the other PCA results). The first and second principal components (PC1, PC2) were considered, accounting for 71% of the total variance of the dataset. Figure 3 displays the location of the peak joint normalized moments in each serve phase (black markers), and the location of the players with each racket (grey markers) in the PC1/PC2 axis system. The peak shoulder flexion moment for the three serve phases and the peak extension moment for the forward swing and follow-through phases contributed to the first component calculation, and the peak shoulder extension moment of the cocking phase to the second. The location of each individual (player and racket) is summarized on figure 3 by the supplementary individuals (white markers). The vertical downward orientation from racket I_L_ to racket I_H_ followed the orientation of PC2, hence suggesting that increased polar moment of inertia of the racket may influence the peak shoulder extension moment during the cocking phase. As a consequence, among the six parameters describing the peak shoulder flexion/extension moment for the three phases of the serve, only the peak extension moment during the cocking phase was used for pairwise comparison. This procedure was applied for each of the eleven remaining datasets, and reduced the original dataset to 10 peak moments and 2 joint power parameters, which are presented in bold in Tables 1 and 2. The polar moment of inertia was deemed to have no effect on the remaining moments/powers.

**Table 1:** Mean (± Standard Error) peak shoulder, elbow and wrist normalized moments (Nm) for rackets with low (I_L_) and high (I_H_) polar moment of inertia during the three phases of the tennis serve, with * for significant difference at p≤0.05, and ** for significant difference at p≤0.01.

		Cocking		Forward Swing		Follow-through	
		I_L_	I_H_	I_L_	I_H_	I_L_	I_H_
Shoulder	Flexion	2.8±0.4	2.8±0.4	5.2±0.7	5.4±0.7	1.2±0.4	1.0±0.3
	Extension	**0.8±0.4**	1.5±0.5**	7.6±0.9	7.9±0.8	7.7±1.0	7.6±0.8
	Adduction	1.6±0.5	1.5±0.3	6.1±0.7	5.9±0.7	**3.4±0.6**	2.5±0.5*
	Abduction	**2.3±0.5**	4.0±0.8*	2.7±0.3	2.3±0.4	4.7±0.5	4.2±0.4
	Internal rotation	4.3±0. 2	4.2±0.2	5.8±0.3	5.9±0.3	1.6±0.4	1.0±0.6
	External rotation	0.7±0.2	1.0±0.4	1.9±0.3	1.9±0.3	4.3±0.7	3.6±0.2
Elbow	Flexion	0.8±0.1	1.1±0.2	1.6±0.5	1.2±0.5	2.2±0.4	1.9±0.3
	Extension	**1.5±0.3**	1.7±0.2**	1.8±0.2	1.9±0.2	1.6±0.4	1.2±0.2
	Varus	4.4±0.3	4.2±0.2	6.0±0.4	6.1±0.4	1.0±0.3	0.6±0.3
	Valgus	**0.9±0.3**	2.0±0.6*	4.9±0.6	5.1±0.6	5.3±0.5	5.3±0.5
	Pronation	0.9±0.1	0.9±0.1	1.2±0.2	1.0±0.2	**1.1±0.2**	0.7±0.1*
	Supination	**0.6±0.1**	1.1±0.2*	0.8±0.2	0.7±0.2	1.2±0.2	1.0±0.1
Wrist	Flexion	1.6±0.3	1.6±0.4	2.9±0.5	2.9±0.5	0.9±0.2	0.5±0.1
	Extension	0.5±0.1	1.1±0.4	**2.8±0.6**	3.3±0.6*	3.6±0.4	3.9±0.4
	Radial deviation	1.5±0.3	1.8±0.3	**2.1±0.4**	2.5±0.4**	2.0±0.3	1.8±0.5
	Ulnar deviation	0.8±0.1	0.9±0.1	1.0±0.3	0.7±0.4	1.3±0.2	1.1±0.2
	Internal rotation	1.6±0.1	1.7±0.2	2.1±0.1	2.2±0.1	1.2±0.2	1.1±0.3
	External rotation	0.5±0.1	0.9±0.3	0.2±0.2	0.3±0.2	**1.5±0.2**	1.2±0.1*

**Table 2:** Mean (± Standard Error) shoulder, elbow and wrist normalized positive and negative powers (W) for rackets with low (I_L_) and high (I_H_) polar moment of inertia during the three phases of the tennis serve, with * for significant difference at p≤0.05, and ** for significant difference at p≤0.01.

		Cocking		Forward swing		Follow-through	
		I_L_	I_H_	I_L_	I_H_	I_L_	I_H_
Shoulder	positive power	3.4±0.7	3.6±0.8	27.7±6.3	26.8±5.6	16.7±0.9	16.8±1.5
	negative power	1.3±0.1	1.5±0.1	23.5±2.9	24.6±3.5	6.6±2.2	4.5±1.2
Elbow	positive power	0.8±0.2	0.9±0.2	9.2±2.5	9.8±2.6	3.5±0.7	4.2±1.0
	negative power	1.1±0.1	1.2±0.1	**17.9±1.8**	19.6±1.9**	5.7±0.8	4.6±0.9
Wrist	positive power	0.8±0.1	0.9±0.2	23.7±3.2	25.4±1.6	12.5±1.2	12.8±0.5
	negative power	0.2±0.1	0.2±0.1	**22.6±2.9**	24.2±2.7*	2.1±0.5	1.2±0.3

Regarding peak shoulder moments (Table 1), significantly higher peak extension and abduction moments during the cocking (*d* = 0.68, medium-to-large effect, p = 0.04, and *d* = 0.80, large effect, p = 0.03, respectively), and significantly lower peak adduction moment during the follow-through were found for I_H_ than for I_L_ (*d* = 0.84, large effect, p = 0.02). At the elbow joint, significantly higher peak extension, valgus and supination moments during the cocking (*d* = 1.21, large effect, p = 0.005; *d* = 0.78, large effect, p = 0.03, and *d* = 0.80, large effect, p = 0.03, respectively), and significantly lower peak pronation moments during the follow-through (*d* = 0.85, large effect, p = 0.02) were observed for I_L_ than for I_H_ (Table 1). At the wrist joint, significantly higher peak extension and radial deviation moments during the forward swing (*d* = 0.81, large effect, p = 0.03, and *d* = 1.43, large effect, p = 0.002, respectively), and significantly lower peak external rotation moments during the follow-through (*d* = 0.66, medium-to-large effect, p = 0.05) were observed for I_L_ than for I_H_ (Table 1).

Concerning joint powers (Table 2), mean negative elbow and wrist normalized powers were significantly lower for I_L_ compared with I_H_ (Table 2) during the forward swing phase (*d* = 1.18, large effect, p = 0.006, and *d* = 0.77, large effect, p = 0.03, respectively).

## Discussion

The aim of this study was to investigate the effects of the racket polar moment of inertia on dominant upper limb joint loads during the tennis serve. The main results show that, during the cocking phase, the peak shoulder extension and abduction, as well as elbow extension, valgus and supination normalized moments significantly increased when the racket polar moment of inertia increased. During the forward swing phase, the peak wrist extension and radial deviation normalized moments significantly increased with an increase in polar moment of inertia. During the follow-through phase, the peak shoulder adduction, elbow pronation and wrist external rotation normalized moments significantly decreased with an increase in polar moment of inertia. Finally, during the forward swing phase, the elbow and wrist negative normalized joint powers were significantly greater when the racket with a high polar moment of inertia was used. These findings provide coaches, players and scientists with new insights on the relationship between racket specifications and upper limb loads, which may be helpful in improving serve performance and for injury prevention.

The racket pre-impact resultant velocities and ball post-impact velocities were slightly lower than those reported in the literature for amateur competition male tennis players [6], [22], [23]. These differences may be explained by our experimental instructions, aiming to achieve similar post-impact ball velocities whatever the racket conditions, while players were instructed to serve at maximal ball velocity in previous studies [6], [22], [23]. Methodological differences could also explain the lower velocities in our study. Indeed, Reid et al. [22] reported the resultant velocity of the marker on the racket tip, which is faster than the resultant velocity of the center of the racket face, calculated in this study. Nevertheless, ball and racket velocities were similar in the two racket conditions, indicating both that the tennis players involved in this study complied with the experimental instructions, and that altering the polar moment of inertia has no effect on racket/ball velocity. Moreover, the duration of the serve phases was in line with those reported by the literature [24], and remained similar in the two racket conditions. The similarities in velocities and phase durations imply that the kinematic patterns remained stable whatever the racket used in the tennis serve. Furthermore, the peak normalized moments (Table 1) were close to those reported by Elliott et al. [10] for peak shoulder internal rotation, elbow varus and flexion normalized moments, while they remained well below those of Martin et al.'s study [6]. For the other orientations and joints, our moment values are difficult to compare to those of the literature. Firstly, in accordance with the recommendations of Bonnefoy et al. [17], the peak shoulder moments in this study were expressed using a different decomposition (XZY) to that used by Elliott et al. [10]. This methodological choice facilitated the clinical and anatomical interpretation of the joint moment components [25], [26]. Secondly, previous work delimited the analyses of net joint moments to a particular axis, joint or phase of the serve. In order to provide a more comprehensive description of the effects of the racket's polar moment of inertia on upper limb loading, it was necessary to examine all of the degrees of freedom at each joint throughout the entire duration of the service action. As such, the Principal Components Analyses were helpful to find the axes, joints and serves phases of interest.

The tennis serve has been known to cause both pain and injury to tennis players of all levels of skill [27]. This potential for musculoskeletal injury is related to the power and acceleration required in a very short time [11], the repetitive nature of these overhead mechanics [10], and the considerable joint loads, especially at the upper limb joints [10], [11], [28]. Net joint moments during the tennis serve are used to predict potentially injurious behavior by associating peak joint moments and overuse injury [6]. It might appear obvious that racket characteristics, especially the polar moment of inertia, are key to minimizing peak joint moments, and thus reducing the potential for injury of the tennis serve; yet no previous study has focused on the effects of the racket polar moment of inertia during the tennis serve. During the terminal point of the cocking phase, shoulder external rotation is driven by inertial lag and stores elastic energy in the passive structures of the shoulder [7]. The elbow flexes to position the racket parallel to and pointing down the spine. This phase is known to generate high values for shoulder internal rotation and horizontal adduction moments, elbow varus and wrist flexion moments [6], [10]. Here, these particular joint moments were unaffected by changes in the polar moment of inertia of the tennis racket. However, the increased polar moment of inertia resulted in larger shoulder extension and abduction moments, as well as elbow extension and supination moments (Table 1). It could therefore be hypothesized that these increased joint moments and the large number of repetitions may contribute to muscular fatigue. In particular, when serving with a racket characterized by a high polar moment of inertia, the shoulder abduction moment could apply additional loads on the supraspinatus muscle in order to abduct the upper limb, and thus to maintain the congruence between the humeral head and glenoid cavity of the scapula. When extended to the whole of the upper limb, the increase in mean joint power coupled with an increased polar moment of inertia could reflect an additional muscular demand so as to maintain the racket in optimal position during the cocking phase (Table 2). However, as no significant differences were observed between the two rackets, this hypothesis remains to be confirmed. During the cocking phase, the elbow valgus moment also appears to increase with the racket's polar moment of inertia (Table 1). This may apply additional internal loads on the osseous and ligamentous structures of the elbow joint, which could contribute to the abutment of the posteromedial olecranon on the medial wall of the olecranon fossa. Specifically, the increased polar moment of inertia of the racket could exacerbate valgus extension overload mechanisms, known to lead to medial elbow injuries [29].

During the forward swing phase of the tennis serve, the dominant upper arm internally rotates vigorously [10], then the forearm pronates and finally the wrist flexes prior to impact [7]. The acceleration of the racket head requires large internal rotation and horizontal adduction moments at the shoulder joint, predominantly generated by the pectoralis major and rotator cuff muscles [11]. Additionally, the high elbow valgus moment can induce damage of the ulnar collateral ligament [11]. At the wrist, most pain occurs because of the repetitive loads induced by internal/external rotation moments [6]. Interestingly, our results showed that the peak moments at the shoulder and elbow joints during the forward swing phase of the serve were unaffected by the increased racket polar moment of inertia (Table 1). However, the increased polar moment of inertia generated significantly higher loads on wrist extension and radial deviation components (Table 1), as well as greater negative joint power at the elbow and wrist (Table 2). This implies that the increase in polar moment of inertia of the tennis racket placed greater demands on the extensor and abductor muscles in order to counteract the movements of wrist flexion and ulnar deviation. A higher polar moment of inertia may then be expected to contribute to overuse injuries in the wrist extensor muscles during the forward swing phase of the tennis serve, which is known to cause lateral epicondylitis pain [11]. Finally, during the follow-through phase of the tennis serve, the racket continues the up- and out- trajectory before it swings across the body [27] to decelerate the shoulder internal rotation, elbow extension and pronation. The deceleration of the racket-upper limb complex generates high horizontal abduction [6] and external rotation moments [22] at the shoulder joint, and places large loads on the rotator cuff muscles in order to maintain the humeral head in the glenoid cavity [11]. According to our results (Table 1), these moment components remained similar in both racket conditions. Additionally, an increased polar moment was associated with decreased peak shoulder adduction and elbow pronation moments, suggesting a reduced need for these deceleration actions. As this reduction was not concomitant with decreased joint power (Table 2) and as the wrist external rotation moment was also reduced in the condition featuring a high polar moment of inertia, it could be hypothesized that increasing the polar moment of inertia may reduce the loads applied on the osseous and ligamentous structures of the upper limb during the follow-through phase of the tennis serve. However, this beneficial effect should be nuanced, because the peak moment values attained during the follow-through remained low compared with those observed during the cocking and forward swing phases (Table 1).

This study presents some limitations that warrant discussion. Aside from the traditional issues relating to motion capture, the small sample size limits the immediate generalizability of these findings, which are also limited to male players and cannot be extended to specific serve types. Another limitation is the lack of markers on the player's lower limbs, making it impossible to break the cocking phase down into a preparation phase and a lower limb propulsion phase. However, the findings remain informative, as this study is the first to examine how the racket's polar moment of inertia affects upper extremity loading in tennis players.

The current findings suggest that a racket with a larger polar moment of inertia induces greater loads on five peak moment components at the shoulder and elbow joints during the cocking phase, and on two components at the wrist joint during the forward swing phase, of the tennis serve. Conversely, a higher racket polar moment of inertia resulted here in reduced loads on three moment components at the three joints of the dominant upper limb that are associated with decelerating the upper limb – racket complex during the follow-through phase of the tennis serve. Although the etiology of overuse injuries is multifactorial, the findings of this study suggest that an increased racket polar moment of inertia could contribute to excessive joint moments in the upper limb joints during the tennis serve, in particular during the cocking and forward swing phases. Therefore, although rackets with a larger polar moment of inertia may better accommodate off-center impacts, they appear more likely to increase loading at the upper extremity joints and increase injury risk therein. Future work should attempt to replicate and confirm these findings, extending the investigation to the temporal patterns of peak moment occurrence. It would also be interesting to investigate whether modifications to either (1) other racket parameters (e.g. mass, strings, or other inertial characteristics), or (2) mechanics (e.g. lower limb drive, grip, ball toss etc.) can offset the increases in upper extremity loading noted in this study.

## Supporting Information

File S1 **Raw data.**
(XLS)

File S2 **Results of Principal Component Analysis for peak shoulder abduction/adduction, shoulder internal/external rotation, elbow flexion/extension, elbow varus/valgus, elbow pronation/supination, wrist flexion/extension, wrist radial/ulnar deviation, wrist internal/external rotation normalized moments, as well as mean shoulder, elbow and wrist joint power.**
(DOCX)
