# Supplementary material for: Effects of the Racket Polar Moment of Inertia on Dominant Upper Limb Joint Moments during Tennis Serve
Source: PLoS One. 2014 Aug 12;9(8):e104785. doi: 10.1371/journal.pone.0104785 (PMC4130553; doi:10.1371/journal.pone.0104785)
Supplement: File S2 — Results of Principal Component Analysis for peak shoulder abduction/adduction, shoulder internal/external rotation, elbow flexion/extension, elbow varus/valgus, elbow pronation/supination, wrist flexion/extension, wrist radial/ulnar deviation, wrist internal/external rotation normalized moments, as well as mean shoulder, elbow and wrist joint power. (DOCX) [file pone.0104785.s002.docx]

**Results of PCA**


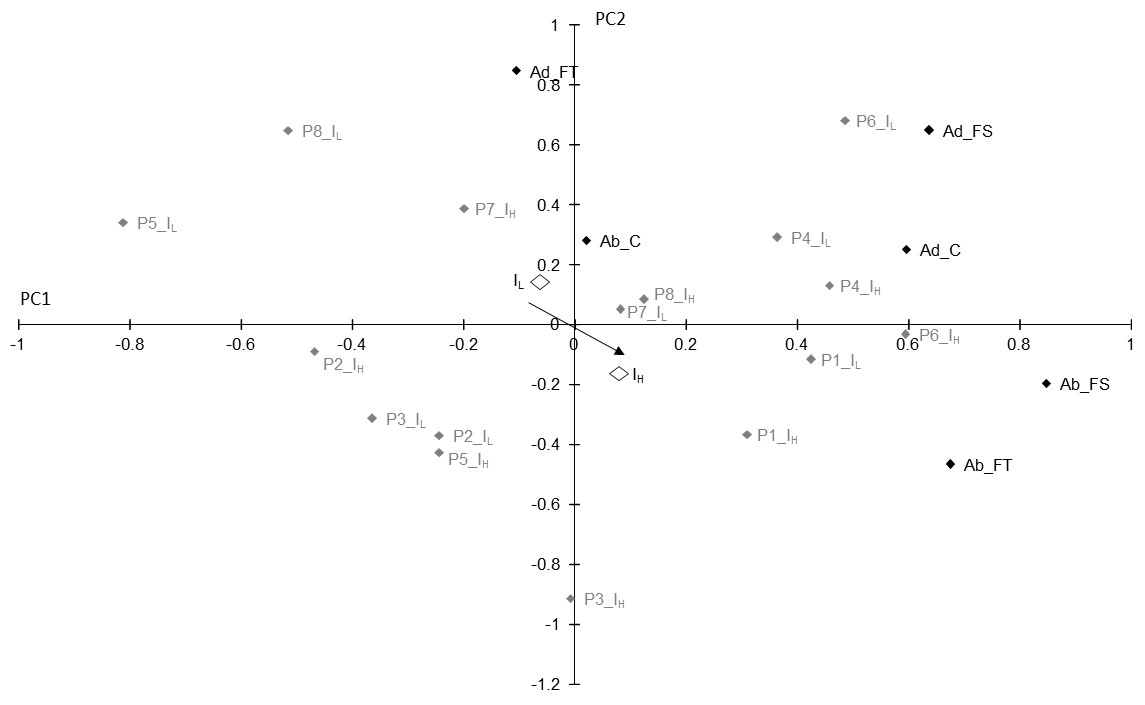


Figure S1: Principal Component 1 (PC1) / Principal Component 2 (PC2) axis system diagram of the peak shoulder Abduction (Ab_) / Adduction (Ad_) normalized moments (black markers) during the cocking (C), forward swing (FS) and follow-through (FT) phases. PC1 explains 33% of the dataset variance and PC2 26%. The individuals are presented by grey markers with PX_I_L_ and PX_I_H_ (X ranged from 1 to 8 according to each player), and the supplementary individuals, summarizing the behavior of I_L_ and I_H_, are displayed by white markers, with I_L_ for the racket with a low polar inertial moment and I_H_ for the racket with a high polar inertial moment. The arrow indicates the main orientation from I_L_ to I_H_.


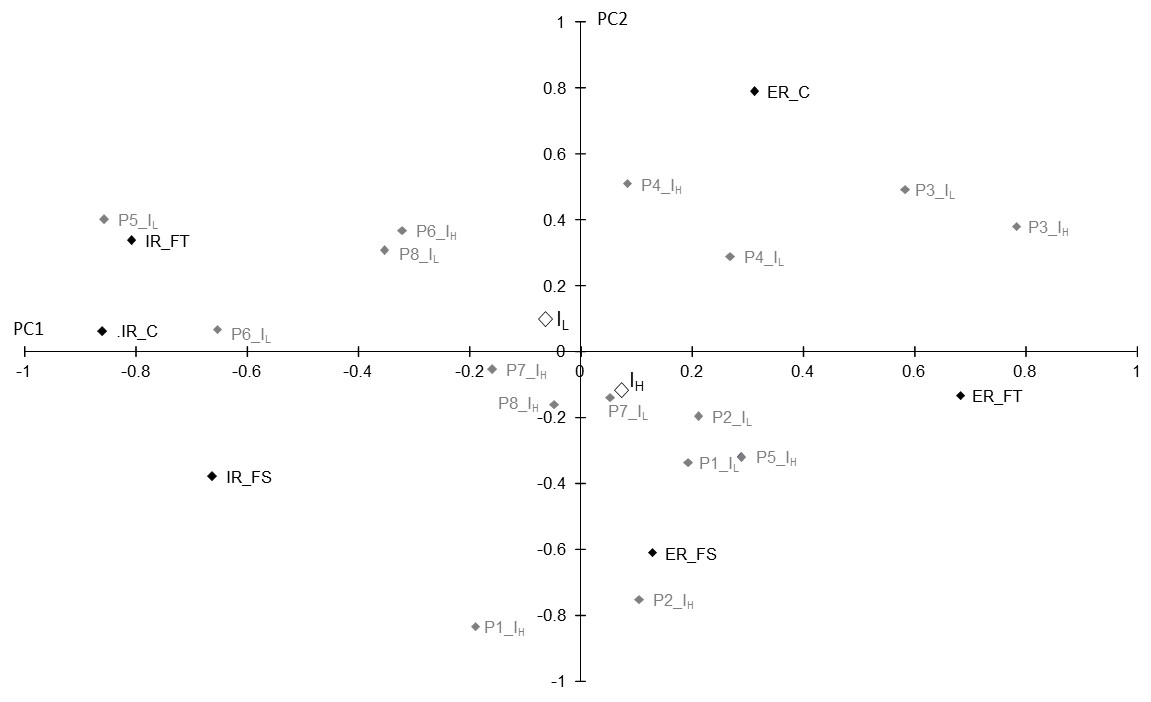


Figure S2: Principal Component 1 (PC1) / Principal Component 2 (PC2) axis system diagram of the peak shoulder Internal (IR_) / External Rotation (ER_) normalized moments (black markers) during the cocking (C), forward swing (FS) and follow-through (FT) phases. PC1 explains 40% of the dataset variance and PC2 21%. The individuals are presented by grey markers with PX_I_L_ and PX_I_H_ (X ranged from 1 to 8 according to each player), and the supplementary individuals, summarizing the behavior of I_L_ and I_H_, are displayed by white markers, with I_L_ for the racket with a low polar inertial moment and I_H_ for the racket with a high polar inertial moment.


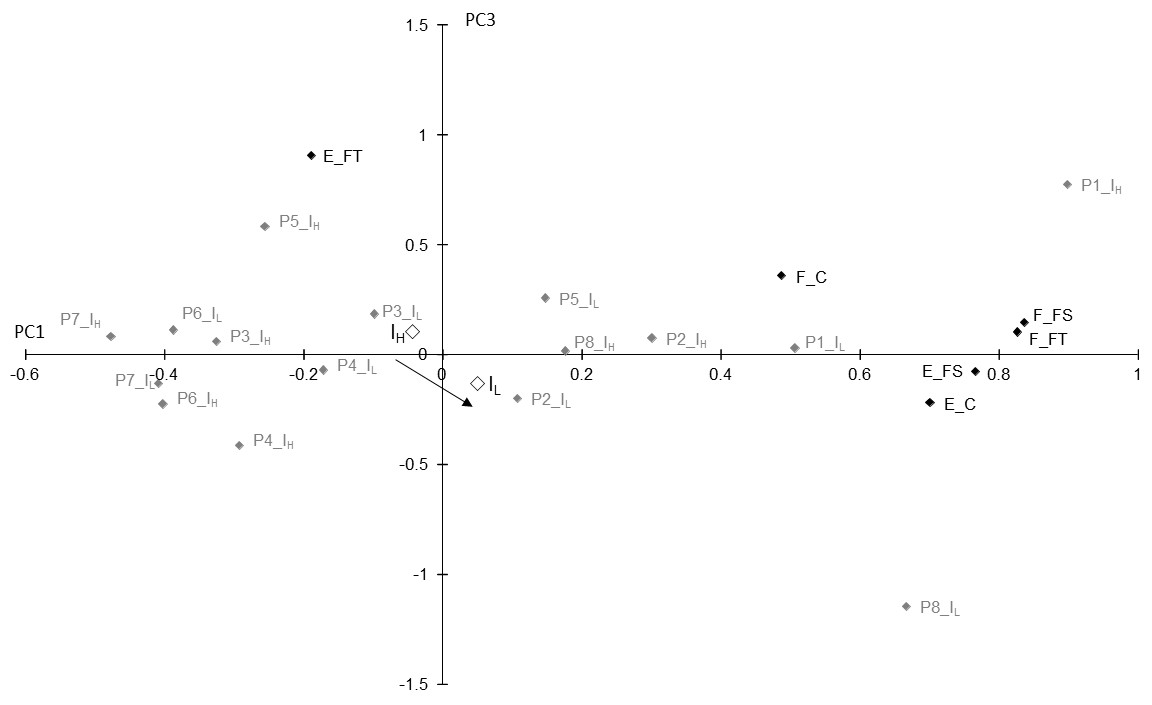


Figure S3: Principal Component 1 (PC1) / Principal Component 3 (PC3) axis system diagram of the peak elbow Flexion (F_) / Extension (E_) normalized moments (black markers) during the cocking (C), forward swing (FS) and follow-through (FT) phases. PC1 explains 46% of the dataset variance and PC3 17%. The individuals are presented by grey markers with PX_I_L_ and PX_I_H_ (X ranged from 1 to 8 according to each player), and the supplementary individuals, summarizing the behavior of I_L_ and I_H_, are displayed by white markers, with I_L_ for the racket with a low polar inertial moment and I_H_ for the racket with a high polar inertial moment. The arrow indicates the main orientation from I_L_ to I_H_.


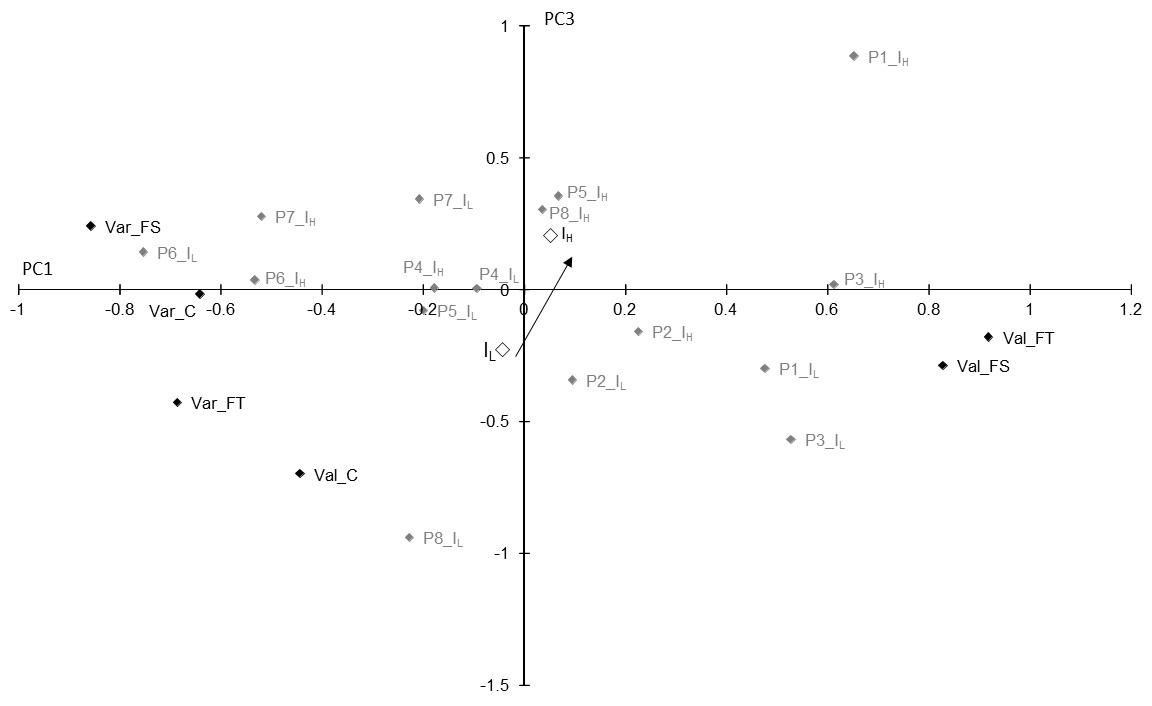


Figure S4: Principal Component 1 (PC1) / Principal Component 3 (PC3) axis system diagram of the peak elbow Varus (Var_) / Valgus (Val_) normalized moments (black markers) during the cocking (C), forward swing (FS) and follow-through (FT) phases. PC1 explains 56% of the dataset variance and PC3 14%. The individuals are presented by grey markers with PX_I_L_ and PX_I_H_ (X ranged from 1 to 8 according to each player), and the supplementary individuals, summarizing the behavior of I_L_ and I_H_, are displayed by white markers, with I_L_ for the racket with a low polar inertial moment and I_H_ for the racket with a high polar inertial moment. The arrow indicates the main orientation from I_L_ to I_H_.


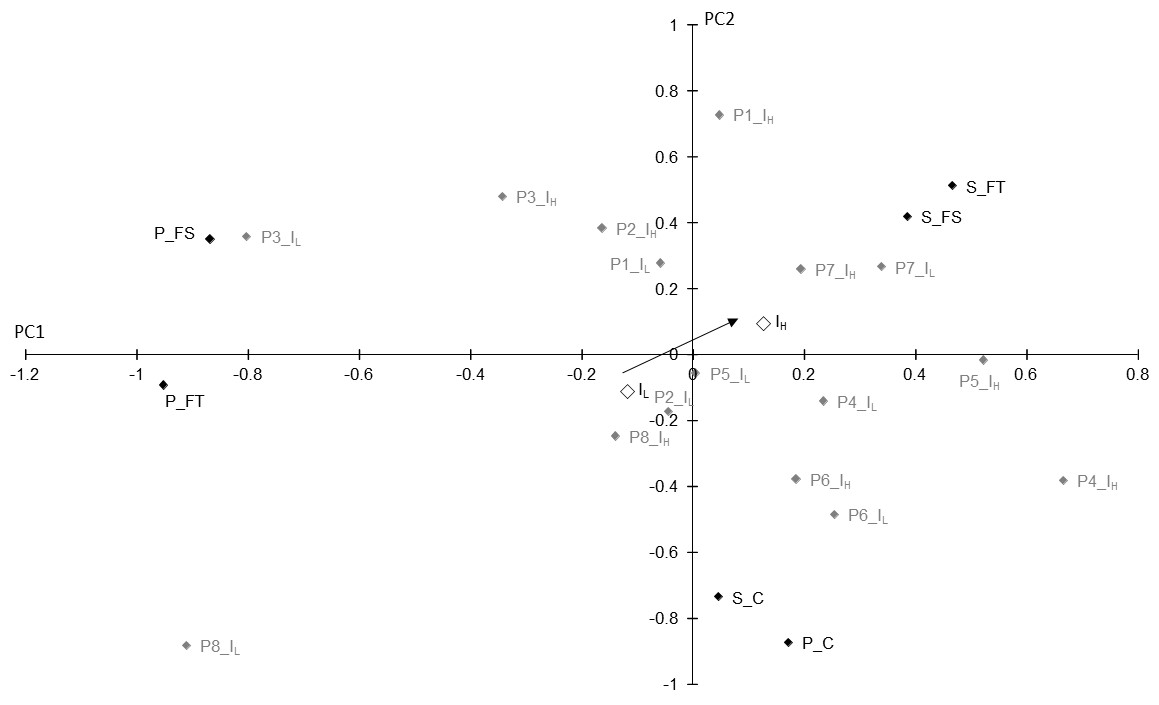


Figure S5: Principal Component 1 (PC1) / Principal Component 2 (PC2) axis system diagram of the peak elbow Pronation (P_) / Supination (S_) normalized moments (black markers) during the cocking (C), forward swing (FS) and follow-through (FT) phases. PC1 explains 34% of the dataset variance and PC2 31%. The individuals are presented by grey markers with PX_I_L_ and PX_I_H_ (X ranged from 1 to 8 according to each player), and the supplementary individuals, summarizing the behavior of I_L_ and I_H_, are displayed by white markers, with I_L_ for the racket with a low polar inertial moment and I_H_ for the racket with a high polar inertial moment. The arrow indicates the main orientation from I_L_ to I_H_.


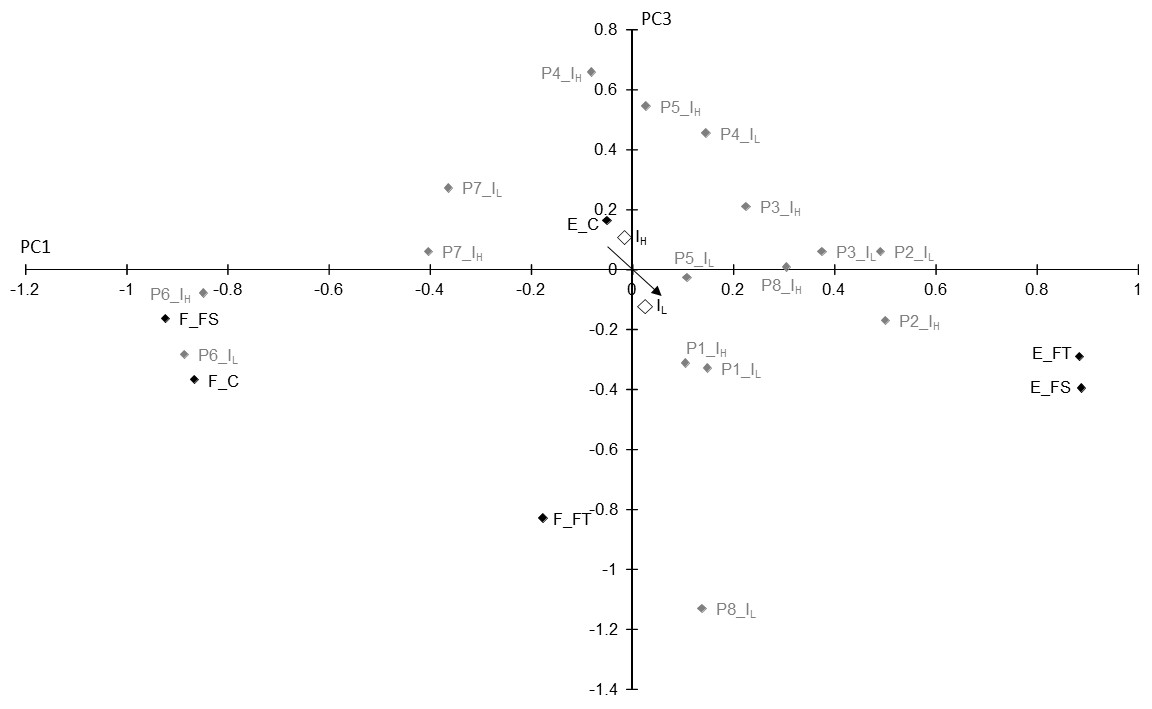


Figure S6: Principal Component 1 (PC1) / Principal Component 3 (PC3) axis system diagram of the peak wrist Flexion (F_) / Extension (E_) normalized moments (black markers) during the cocking (C), forward swing (FS) and follow-through (FT) phases. PC1 explains 53% of the dataset variance and PC3 19%. The individuals are presented by grey markers with PX_I_L_ and PX_I_H_ (X ranged from 1 to 8 according to each player), and the supplementary individuals, summarizing the behavior of I_L_ and I_H_, are displayed by white markers, with I_L_ for the racket with a low polar inertial moment and I_H_ for the racket with a high polar inertial moment. The arrow indicates the main orientation from I_L_ to I_H_.


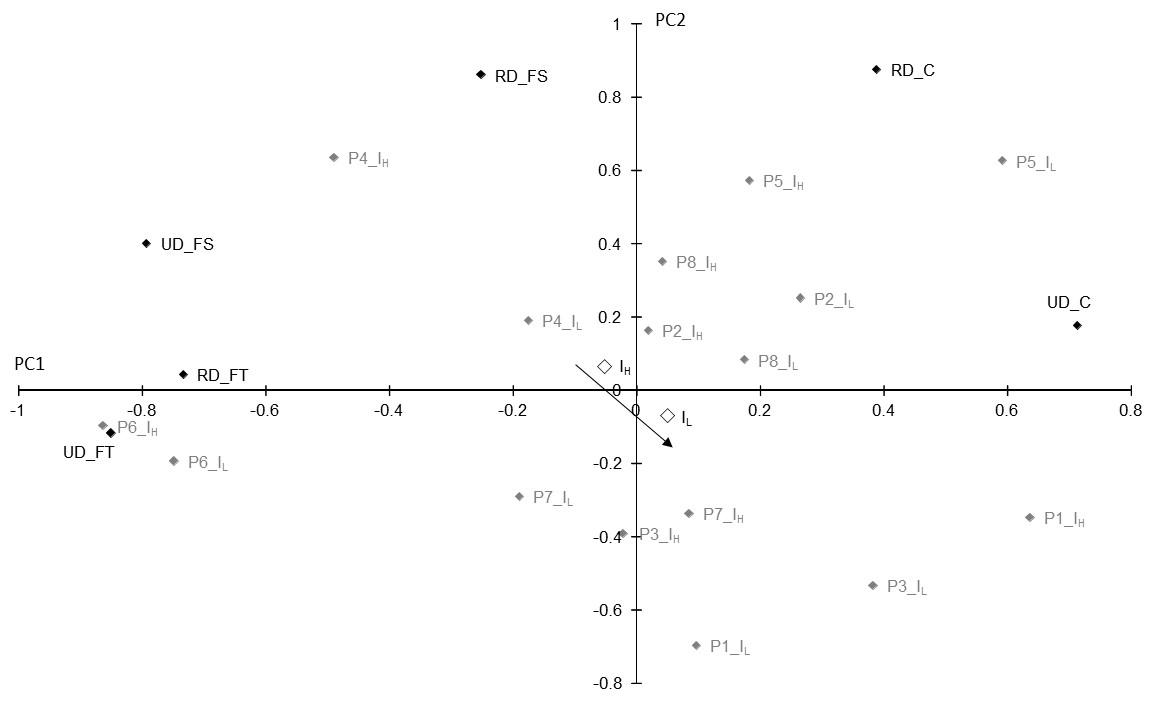


Figure S7: Principal Component 1 (PC1) / Principal Component 2 (PC2) axis system diagram of the peak wrist Radial Deviation (RD_) / Ulnar Deviation (UD_) normalized moments (black markers) during the cocking (C), forward swing (FS) and follow-through (FT) phases. PC1 explains 43% of the dataset variance and PC2 29%. The individuals are presented by grey markers with PX_I_L_ and PX_I_H_ (X ranged from 1 to 8 according to each player), and the supplementary individuals, summarizing the behavior of I_L_ and I_H_, are displayed by white markers, with I_L_ for the racket with a low polar inertial moment and I_H_ for the racket with a high polar inertial moment. The arrow indicates the main orientation from I_L_ to I_H_.


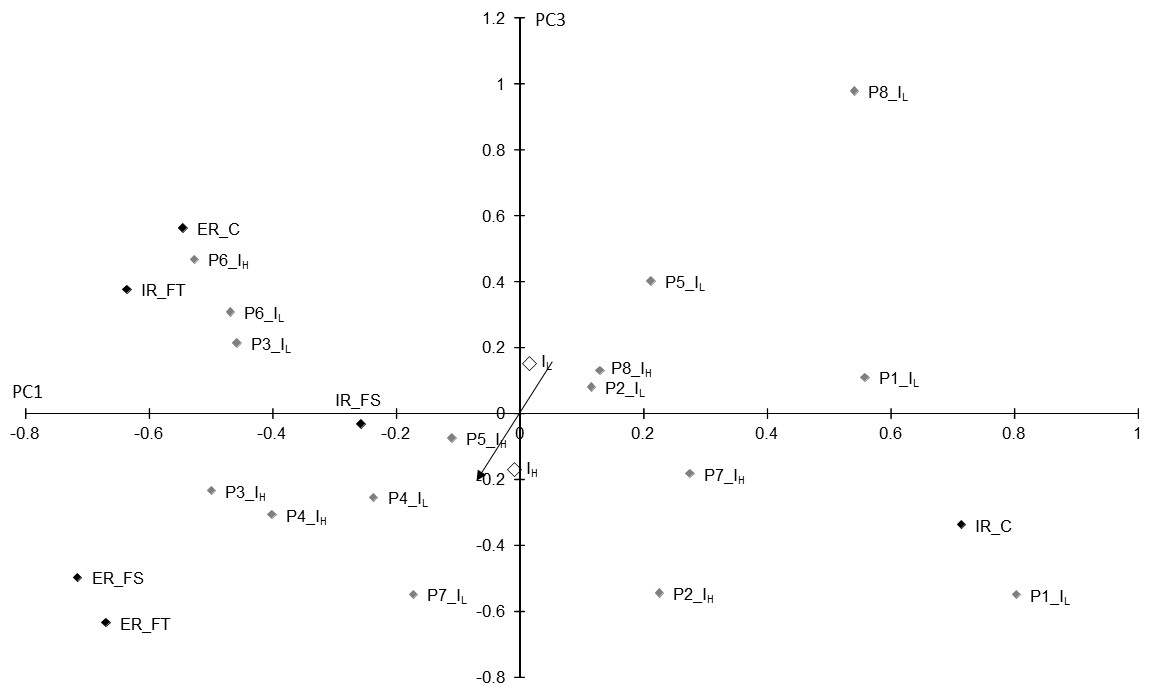


Figure S8: Principal Component 1 (PC1) / Principal Component 3 (PC3) axis system diagram of the peak wrist Internal Rotation (IR_) / External Rotation (ER_) normalized moments (black markers) during the cocking (C), forward swing (FS) and follow-through (FT) phases. PC1 explains 37% of the dataset variance and PC3 20%. The individuals are presented by grey markers with PX_I_L_ and PX_I_H_ (X ranged from 1 to 8 according to each player), and the supplementary individuals, summarizing the behavior of I_L_ and I_H_, are displayed by white markers, with I_L_ for the racket with a low polar inertial moment and I_H_ for the racket with a high polar inertial moment. The arrow indicates the main orientation from I_L_ to I_H_.


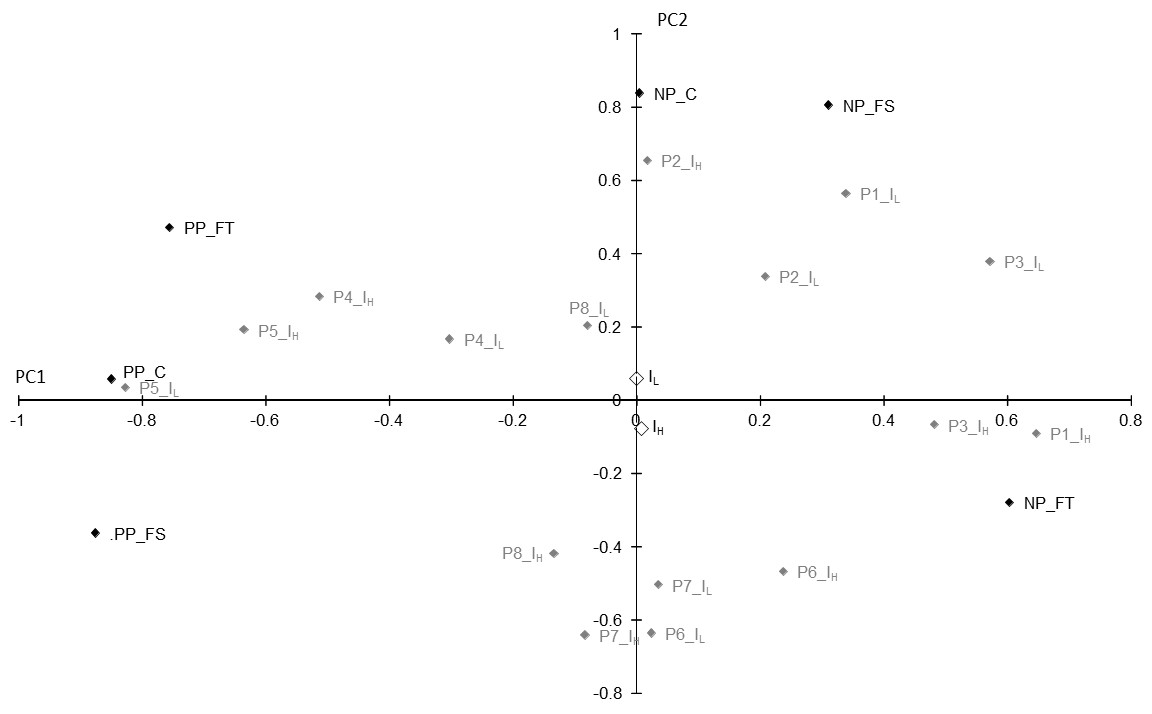


Figure S9: Principal Component 1 (PC1) / Principal Component 2 (PC) axis system diagram of the mean shoulder positive power (PP_) / Negative Power (NP_) normalized moments (black markers) during the cocking (C), forward swing (FS) and follow-through (FT) phases. PC1 explains 42% of the dataset variance and PC2 30%. The individuals are presented by grey markers with PX_I_L_ and PX_I_H_ (X ranged from 1 to 8 according to each player), and the supplementary individuals, summarizing the behavior of I_L_ and I_H_, are displayed by white markers, with I_L_ for the racket with a low polar inertial moment and I_H_ for the racket with a high polar inertial moment.


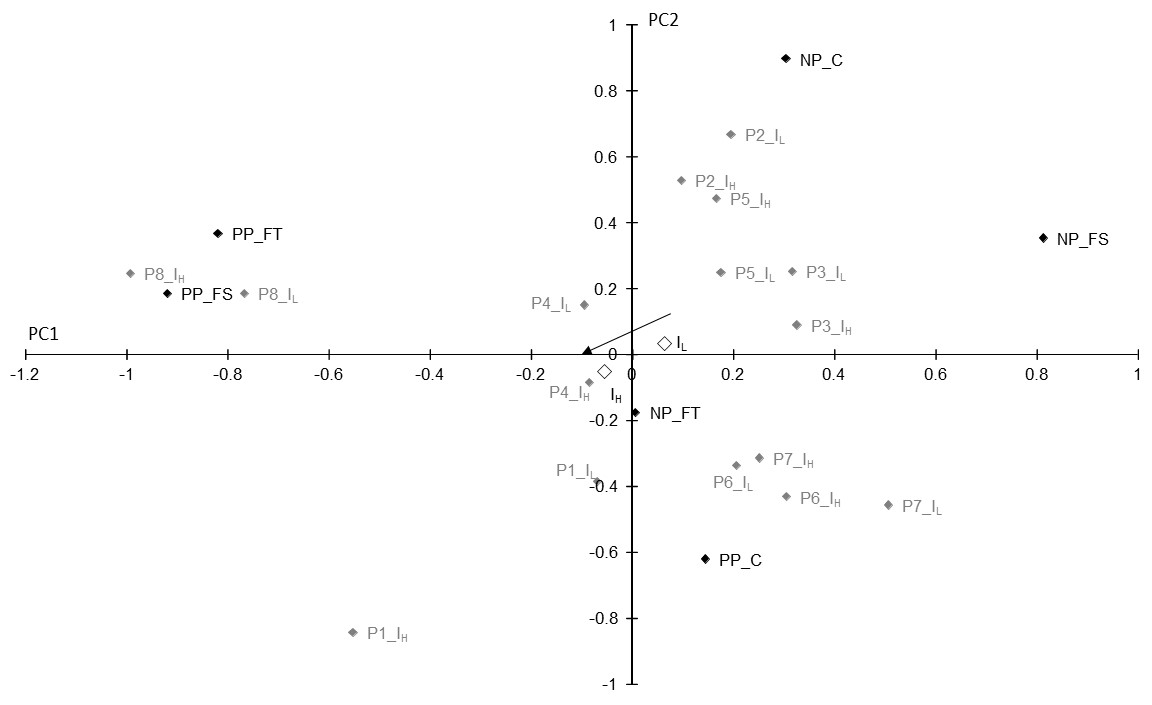


Figure S10: Principal Component 1 (PC1) / Principal Component 2 (PC) axis system diagram of the mean elbow positive power (PP_) / Negative Power (NP_) normalized moments (black markers) during the cocking (C), forward swing (FS) and follow-through (FT) phases. PC1 explains 38% of the dataset variance and PC2 25%. The individuals are presented by grey markers with PX_I_L_ and PX_I_H_ (X ranged from 1 to 8 according to each player), and the supplementary individuals, summarizing the behavior of I_L_ and I_H_, are displayed by white markers, with I_L_ for the racket with a low polar inertial moment and I_H_ for the racket with a high polar inertial moment. The arrow indicates the main orientation from I_L_ to I_H_.


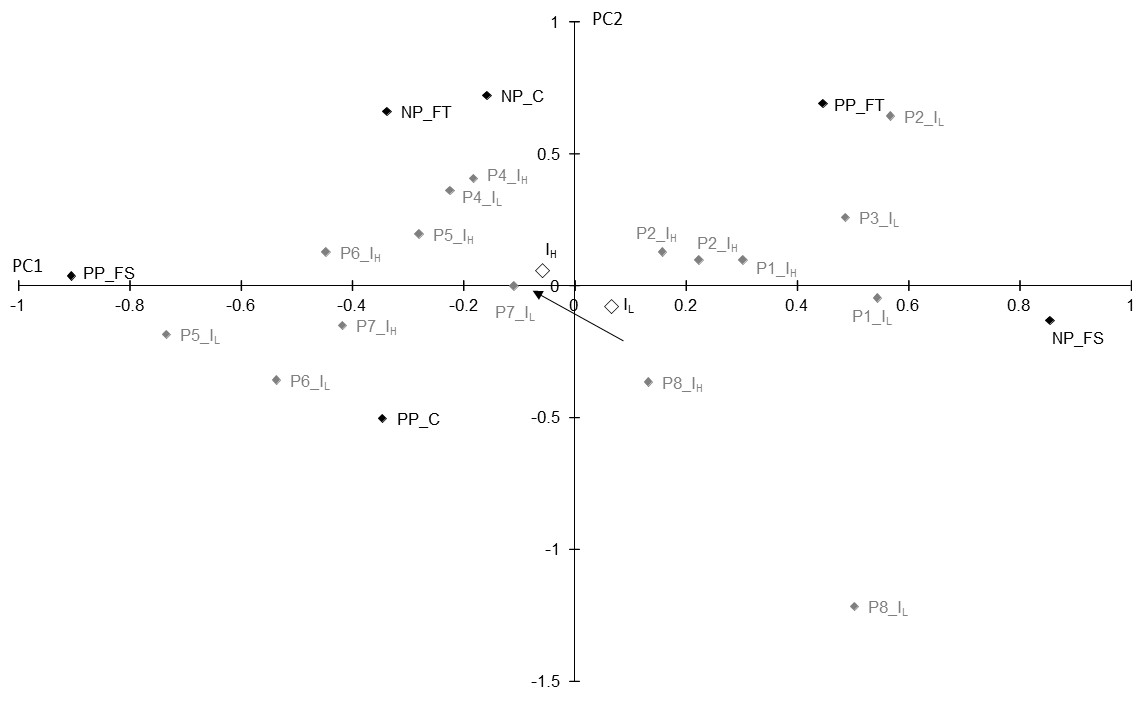


Figure S11: Principal Component 1 (PC1) / Principal Component 2 (PC) axis system diagram of the mean wrist positive power (PP_) / Negative Power (NP_) normalized moments (black markers) during the cocking (C), forward swing (FS) and follow-through (FT) phases. PC1 explains 33% of the dataset variance and PC2 28%. The individuals are presented by grey markers with PX_I_L_ and PX_I_H_ (X ranged from 1 to 8 according to each player), and the supplementary individuals, summarizing the behavior of I_L_ and I_H_, are displayed by white markers, with I_L_ for the racket with a low polar inertial moment and I_H_ for the racket with a high polar inertial moment. The arrow indicates the main orientation from I_L_ to I_H_.
